# Application of the Gait Deviation Index to Study Gait Impairment in Adult Population With Spinal Cord Injury: Comparison With the Walking Index for Spinal Cord Injury Levels

**DOI:** 10.3389/fnhum.2022.826333

**Published:** 2022-04-04

**Authors:** Isabel Sinovas-Alonso, Diana Herrera-Valenzuela, Roberto Cano-de-la-Cuerda, Ana de los Reyes-Guzmán, Antonio J. del-Ama, Ángel Gil-Agudo

**Affiliations:** ^1^International Doctoral School, Rey Juan Carlos University, Madrid, Spain; ^2^Biomechanics and Technical Aids Unit, National Hospital for Paraplegics, Toledo, Spain; ^3^Faculty of Health Sciences, Department of Physical Therapy, Occupational Therapy, Physical Medicine and Rehabilitation, Rey Juan Carlos University, Alcorcón, Spain; ^4^School of Science and Technology, Department of Applied Mathematics, Materials Science, Engineering and Electronic Technology, Rey Juan Carlos University, Móstoles, Spain

**Keywords:** spinal cord injury (SCI), gait impairment, Gait Deviation Index (GDI), three-dimensional (3D) kinematic gait data, walking index for spinal cord injury (WISCI)

## Abstract

The Gait Deviation Index (GDI) is a multivariate measure of overall gait pathology based on 15 gait features derived from three-dimensional (3D) kinematic data. GDI aims at providing a comprehensive, easy to interpret, and clinically meaningful metric of overall gait function. It has been used as an outcome measure to study gait in several conditions: cerebral palsy (CP), post-stroke hemiparetic gait, Duchenne muscular dystrophy, and Parkinson’s disease, among others. Nevertheless, its use in population with Spinal Cord Injury (SCI) has not been studied yet. The aim of the present study was to investigate the applicability of the GDI to SCI through the assessment of the relationship of the GDI with the Walking Index for Spinal Cord Injury (WISCI) II. 3D gait kinematics of 34 patients with incomplete SCI (iSCI) was obtained. Besides, 3D gait kinematics of a sample of 50 healthy volunteers (HV) was also gathered with Codamotion motion capture system. A total of 302 (iSCI) and 446 (HV) strides were collected. GDI was calculated for each stride and grouped for each WISCI II level. HV data were analyzed as an additional set. Normal distribution for each group was assessed with Kolmogorov-Smirnov tests. Afterward, ANOVA tests were performed between each pair of WISCI II levels to identify differences among groups (*p* < 0.05). The results showed that the GDI was normally distributed across all WISCI II levels in both iSCI and HV groups. Furthermore, our results showed an increasing relationship between the GDI values and WISCI II levels in subjects with iSCI, but only discriminative in WISCI II levels 13, 19, and 20. The index successfully distinguished HV group from all the individuals with iSCI. Findings of this study indicated that the GDI is not an appropriate multivariate walking metric to represent the deviation of gait pattern in adult population with iSCI from a normal gait profile when it is compared with the levels of walking impairment described by the WISCI II. Future work should aim at defining and validating an overall gait index derived from 3D kinematic gait variables appropriate for SCI, additionally taking into account other walking ability outcome measures.

## Introduction

The incidence of spinal cord injury (SCI) worldwide is between 250,000 and 500,000 individuals each year (Quadri et al., 2020). In Western European countries traumatic SCI incidence is of 16 to 19.4 new cases per million inhabitants per year (Scivoletto et al., 2017). Walking is usually affected in patients with SCI according to the lesion level and the resulting different levels of muscle paralysis, sensory impairment, spasticity, and the lack of trunk control (Bani et al., 2013). In the field of SCI research, there is an emphasis on the ability to ambulate as a functional outcome and as an indicator of quality of life (Jackson et al., 2008), particularly in individuals with incomplete SCI (iSCI) (Ditunno et al., 2008).

Walking function recovery is tackled through several therapeutic interventions such as surgery, physiotherapy, medications, orthotics, and robotics in which precise evaluation of walking function is mandatory (Scivoletto et al., 2011). Periodic gait measurements can be used to evaluate the response to these therapeutical approaches (McGinley et al., 2009), to assess changes in walking over time, and to discriminate between normal and altered gait (Baker, 2006). In this regard, three-dimensional (3D) kinematic gait analysis can provide useful information to guide rehabilitation interventions to improve walking function of people with traumatic and non-traumatic iSCI (Murphy et al., 2019). Nevertheless, isolated kinematic parameters do not provide a full picture of gait pattern impairment (Guzik and Drużbicki, 2020), and on the other hand, it may be difficult to describe objectively the heterogeneity of the different gait abnormalities present in iSCI and to quantify the degree by which they deviate from normal gait patterns. The Gait Deviation Index (GDI) is a multivariate measure of overall gait pathology based on 15 gait features built upon 3D kinematic data originally designed from a sample of children with cerebral palsy (CP) (Schwartz and Rozumalski, 2008). The GDI is a dimensionless parameter represented as a single score for an individual gait deviation from a normative reference group, which aims at providing a comprehensive, easy to interpret, and clinically meaningful metric of overall gait function.

The usefulness of GDI has been assessed through correlations with clinically-validated gait scales. Concurrent and face validity of GDI was firstly carried out by comparison with the Gillette Functional Assessment Questionnaire walking scale (FAQ) and topographic classifications of CP in children population (Schwartz and Rozumalski, 2008). Later, the relationship between the GDI, Gross Motor Function Measure (GMFM), and Gross Motor Function Classification System (GMFCS) in a representative sample of ambulatory children with CP provided greater validity to the GDI (Molloy et al., 2010). The ability of the GDI to distinguish between GMFCS levels in children with CP in the study developed by (Massaad et al., 2014) concurred with those found by Molloy et al. (2010) and by Schwartz and Rozumalski (2008) for the FAQ. Furthermore, face validity of the GDI in adults with CP was demonstrated by comparing with GMFCS (Maanum et al., 2012), which showed similar distributional properties as those reported in children with CP. The GDI was able to distinguish different levels of gait impairment in adults (Maanum et al., 2012) and children (Schwartz and Rozumalski, 2008; Molloy et al., 2010; Massaad et al., 2014) with CP. However, no correlations have been published between the GDI and other valid walking ability outcome measures commonly used in clinical settings to assess gait variability in adult population with SCI.

The Walking Index for Spinal Cord Injury (WISCI) II is a walking scale specifically developed for iSCI population composed of 21 levels (Dittuno et al., 2001), in which levels are ordered by degree of an individual’s walking impairment, from most impaired to least impaired (Ditunno et al., 2007), integrating a hierarchical order for the use of ambulatory assistive devices (AADs), orthoses, and the physical assistance needed to complete a 10 m walking distance. WISCI II levels differs from self-selected (SS) WISCI, defined as patient’s preferential condition to walk in the community or the household, and maximum WISCI, which is related to the highest level at which a person can safely walk 10 m (Burns et al., 2011). The WISCI II is a valid (Morganti et al., 2005; Ditunno et al., 2007), reliable (Marino et al., 2010; Scivoletto et al., 2014), and responsive (van Hedel et al., 2006) outcome measure to assess walking ability in people with SCI. In our best knowledge, there is no scientific literature which have studied the relationship between the GDI and the WISCI II in adult population with SCI.

The GDI has been used as an outcome measure to study gait in several conditions such as: CP (Schwartz and Rozumalski, 2008; Molloy et al., 2010; Cimolin et al., 2011; Sagawa et al., 2013; Massaad et al., 2014; Wilson et al., 2015; Malt et al., 2016; Ito et al., 2019; Rasmussen et al., 2019), post-stroke hemiparetic gait (Correa et al., 2017; Guzik and Drużbicki, 2020), Duchenne muscular dystrophy (Sienko Thomas et al., 2010), Parkinson’s disease (Galli et al., 2012; Speciali et al., 2013), arthritis (Broström et al., 2013; Esbjörnsson et al., 2014; Rosenlund et al., 2016; Kobsar et al., 2019; Bazarnik-Mucha et al., 2020), lower limb amputations (Eshraghi et al., 2014; Kark et al., 2016), degenerative spinal pathologies (Mar et al., 2019; Trivedi et al., 2021; Zhou et al., 2021), diverse genetic (Ito et al., 2020; Mindler et al., 2020) and congenital disorders (Eriksson et al., 2015; Garman et al., 2019), and even the effect of the COVID-19 on physical function (Ito et al., 2021), among others. A recently published article by Hwang et al. (2021) used the GDI as a way to quantify and characterize gait patterns in ambulatory children and adolescents with transverse myelitis, whose gait showed moderate kinematic deviations from normal gait pattern. Nevertheless, to date, no work has been published regarding the validity of the GDI in population with SCI.

Joint kinematics and spatiotemporal gait parameters differ between adult and child population due to the maturation and aging processes of the gait, associated to the neuromuscular development and the changes in strength that occur during adolescence and adulthood (Cupp et al., 1999; Ganley and Powers, 2005). In this regard, it is necessary to consider the functional differences of the gait pattern in relation to the mature stage in people with SCI.

The aim of the present study was to evaluate the relationship between the GDI and WISCI II levels in adult population with iSCI. Our hypothesis was that the most altered gait kinematics of people with iSCI, reflected by GDI values below 100, would be associated with lower scores of the WISCI II.

## Materials and Methods

### Study Design

An observational retrospective study was conducted on a database of 3D kinematic gait analysis of adult population composed by patients with iSCI and healthy volunteers (HV) gathered between August 2019 and July 2021 at the Biomechanics and Technical Aids Unit of the National Hospital for Paraplegics of Toledo (Spain). All the individuals recruited for the study signed informed consent to participate in the study. According to the Declaration of Helsinki, all participants were informed about the purpose and course of the study, and about their rights to withdraw from the study. The study protocol was reviewed and approved by the Local Ethics Committee of University Hospital Complex of Toledo, Spain.

### Participants

Patients included in the study met the following inclusion criteria: (i) subjects aged 16 years or over; (ii) having suffered a SCI regardless of the etiology (traumatic or non-traumatic), time since injury onset, and neurological level of injury (NLI); (iii) classified as C, D, or E by the American Spinal Injury Association (ASIA) Impairment Scale (AIS) (Kirshblum et al., 2011); (iv) with the ability of walking 10 m independently with any type of external assistance required (orthoses, crutches or canes); (v) with SS WISCI II levels collected; and (vi) capacity to be informed and give consent to participate in the study. Patients from the database were excluded of the study if they followed one of the different conditions: (i) having suffered from rheumatic, orthopedic, or other neurological disorders outside of SCI that affected gait; (ii) need for support in parallel bars, walker and/or physical assistance required of one or two people to walk 10 m safely; (iii) psychiatric or cognitive conditions that may have interfered with the performance of the gait analysis.

Based on the medical history reported by HV in the recruitment process, they were excluded if they experienced musculoskeletal or neurological disorders that affected gait. 3D kinematic data acquired from HV were used to calculate an average normal value of gait kinematics and hence to calculate the deviation from normal gait pattern for each patient, in essence, the GDI.

### Experimental Protocol

**FIGURE 1:**
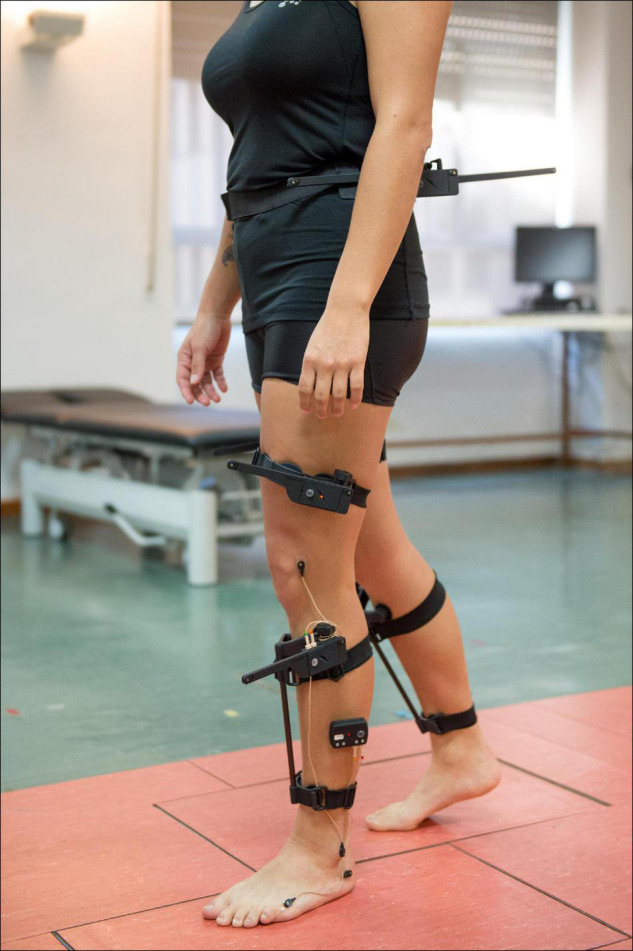
Placement of the 22 active markers of Codamotion motion capture system on the lower limbs of an individual from HV group.

3D kinematic gait data were obtained with Codamotion motion capture system (Charnwood Dynamics, Ltd., United Kingdom), comprised of 22 active markers placed on the lower limbs (Figure 1), three scanners, and two Kistler force platforms embedded in a 10-m walkway. Markers were positioned on the following anatomical references: sacrum (two lateral markers), anterior superior iliac spines (ASIS), posterior superior iliac spines (PSIS), lateral surface of the thighs (anterior and posterior femur markers), lateral femoral condyles, lateral surface of the legs (anterior and posterior tibia markers), lateral malleoli, calcaneus (posterior lateral heels), and fifth metatarsal heads. Marker trajectories were collected at a sampling frequency of 200 Hz. A 3D skeletal model was created for each individual based on markers placement and anthropometric measures taken for each subject, which included: weight, height, pelvis width and depth, knees and ankles width. Subjects were informed to walk naturally at their SS speed with the minimum external assistance required -canes, crutches, and/or orthoses-. A valid stride was considered as the one in which each foot was on a different force platform. Five complete gait cycles or three complete cycles in those individuals with SCI who were not able to get five valid cycles were collected, time-normalized and averaged. A total of 302 and 446 strides were collected for the group with iSCI and the HV group, respectively. The complete records were then processed using the software for data analysis ODIN v.2.02 (Codamotion Ltd., United Kingdom) to calculate the mean values of 3D kinematic parameters for the gait cycle of the right and left leg, for pelvis, and hip, knee, and ankle joints.

### Data Analysis

The GDI is calculated upon the procedure described in Schwartz and Rozumalski (2008). The index is derived from a set of nine kinematic curves of a single stride: i) pelvic orientation and hip angles in the three planes of space (sagittal, frontal and transversal), (ii) knee flexion and extension, (iii) ankle dorsiflexion and plantar flexion, and (iv) foot progression angle.

In the original study (Schwartz and Rozumalski, 2008), a dataset with more than 6,000 strides of patients with CP was used to calculate a 15-feature basis to account for 98% of the total variation of the whole dataset and to allow to reconstruct the kinematic gait curves with a 98% fidelity on average. This basis allowed to calculate the representation of any kinematic gait curve, by multiplying the basis with the kinematic curves of a stride. Afterward, the Euclidean distance between this kinematic gait curve and the average of a set of healthy control strides were calculated, so that the deviation of a gait pattern from a normal gait profile was represented. Lastly, this value was scaled to improve the interpretability of the index, so that every 10 points of GDI below 100 corresponded to 1 standard deviation (SD) away from the typical gait kinematics, whereas a score ≥ 100 represented a normal gait profile.

The GDI for our sample population was calculated for each stride in both groups, subjects with iSCI and HV group, using the orthonormal basis provided in Schwartz and Rozumalski (2008). HV group data, used as the reference gait pattern to compute the gait deviation, were collected following the same procedure used with the individuals with iSCI. Each 3D kinematic gait analysis was associated to a SS WISCI II level according to the preferential condition to walk declared by the participants with iSCI. GDI data were grouped according to the corresponding WISCI II level and HV group data were considered as an additional set. Normal distribution for each group was assessed with Kolmogorov-Smirnov tests. To facilitate the analysis, a histogram of the GDI data comprised within each WISCI II level was calculated with a normal distribution curve fitted to its mean and SD. Afterward, one-way ANOVA tests were performed between the GDI values of each pair of WISCI II levels to identify differences among groups. *P*-value was set to *p* < 0.05 for all statistical procedures. All the data analysis was performed with Matlab R2019a (The MathWorks, Inc., Natick, MA, United States).

## Results

Thirty-four (*n* = 34) adults with iSCI and fifty (*n* = 50) HV met the inclusion criteria (Table 1). Clinical characteristics of individuals with iSCI are shown in Table 2. The dataset of iSCI sample included the following WISCI II levels: 12, 13, 15, 16, 18, 19, and 20.

**TABLE 1:** Characteristics of individuals recruited for the study.

	HV	iSCI
N	50	34
Gender (M/F)	19/31	26/8
Age (years)	34.6 (15.2)	34.6 (18.0)
Height (cm)	166.6 (8.7)	169.7 (10.7)
Weight (Kg)	69.6 (15.0)	70.0 (15.2)
BMI (kg/m^2^)	24.9 (4.0)	24.2 (4.2)

*Parameters reported as mean (SD). HV, healthy volunteers; iSCI, incomplete spinal cord injury; M, male; F, female; SD, standard deviation; BMI, body mass index.*

**TABLE 2:** Clinical characteristics of individuals with iSCI.

Clinical characteristics	iSCI (*n* = 34)	
NLI	C1-C7	16
	T1-T6	3
	T7-L1	11
	L2-L5	4
AIS	C	4
	D	28
	E	2
Etiology (T/NT)	19T/15NT	
Time since injury (years)	5.7 (7.8)	
SS WISCI II level	17.8 (2.4)	

*Parameters reported as mean (SD). NLI, neurological level of injury; AIS, American spinal injury association impairment scale; T, traumatic; NT, non-traumatic; SS WISCI II, self-selected walking index for spinal cord injury II.*

**FIGURE 2:**
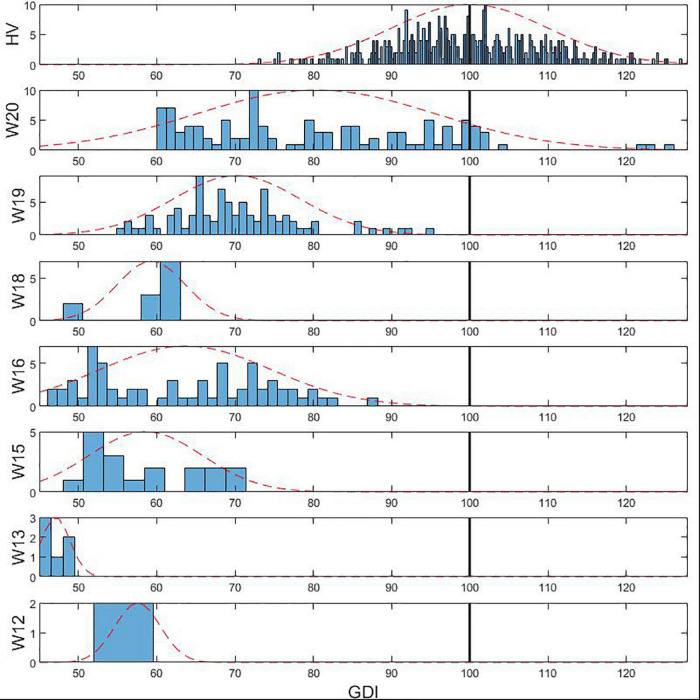
Histograms of GDI values stratified by WISCI II levels in population with iSCI. The dotted line represents the normal distribution curve fitted to the data wihtin each WISCI II level. The vertical black line indicates the HV mean.

The analysis showed that GDI data were normally distributed across all WISCI II levels and also in the HV group. Table 3 presents the number of strides, the range, the mean, and the SD of GDI values comprised in each WISCI II level. Results showed a trend of increasing average GDI values with decreasing level of walking impairment in WISCI II levels 13 to 20 and the HV group, except in level 18, whose average GDI was lower than the average on level 16. This can be easily seen in Figure 2, that shows the histograms of the GDI values stratified by WISCI II levels. Statistically significant differences were found between HV group and all WISCI II levels. Nevertheless, they were only found between WISCI II levels 13, 19, and 20. No statistically significant differences were found between levels under 18 (inclusive), except by level 13. Therefore, the increasing relationship between the GDI values and WISCI II levels was only discriminative in the highest levels in subjects with iSCI (WISCI II 19: 70.2 ± 8.1; WISCI II 20: 80.4 ± 15.2), but not in the lower levels, except in WISCI II level 13 (47.1 ± 1.8).

**TABLE 3:** Descriptive statistics of GDI values (mean and range) within each WISCI II level.

WISCI II levels	*n* strides	Mean (SD)	Range
12 _(13_, _19_, _20, HV)_	2	57.6 (2.9)	55.5-59.6
13 _(12_, _15_, _16_, _18_, _19_, _20, HV)_	6	47.1 (1.8)	45.0-49.2
15 _(13_, _19_, _20, HV)_	18	58.6 (7.2)	49.6-70.7
16 _(13_, _19_, _20, HV)_	65	63.7 (11.0)	46.6-88.2
18 _(13_, _19_, _20, HV)_	12	59.3 (4.4)	49.9-62.6
19 _(12_, _13_, _15_, _16_, _18_, _20, HV)_	87	70.2 (8.1)	52.2-95.3
20 _(12_, _13_, _15_, _16_, _18_, _19, HV)_	112	80.4 (15.2)	60.0-126.0
HV _(12_, _13_, _15_, _16_, _18_, _19_, _20)_	446	100.0 (10.0)	73.1-127.9

*Groups in parentheses indicate statistically significant differences (p < 0.05).*

## Discussion

Our results showed an increasing relationship between the GDI values and WISCI II levels from 13 to 20, and the HV group, except for level 18. Nevertheless, results of the study showed that the application of the GDI only distinguished WISCI II levels 13 (gait assisted with a walker), 19 (gait assisted with a cane), and 20 (no assistance required) from all the other WISCI II levels in adult population with iSCI. The index successfully distinguished all the individuals with iSCI from HV group. For those with WISCI II level 20, GDI values were able to discriminate successfully an impaired gait, even if it did not required any external assistance, from a normal gait pattern. These results does not support previous studies in which WISCI II showed a ceiling effect (Lemay and Nadeau, 2010; Wirz et al., 2010) and a better sensitivity to change in spinal cord injured subjects with more impaired gait compared to those with higher levels of walking function (van Hedel et al., 2006). Regarding ranges of GDI values below WISCI II level 19, except for level 13, results showed an overlap between the different levels, which indicates that the GDI compresses into a small range all WISCI II levels.

Altogether, our results indicate that the GDI was not able to discriminate the functional diversity of adult population with iSCI related to walking impairment defined by WISCI II levels. Therefore, results do not support our hypothesis, built upon the previous correlation analysis of the GDI with other clinical scales used in CP (Schwartz and Rozumalski, 2008; Molloy et al., 2010; Maanum et al., 2012; Massaad et al., 2014). Although more impaired gait patterns, lower GDI values, are associated with lower WISCI II levels, as shown by the stratification, the differences between all levels are not statistically significant. Thus, the GDI is not a valid metric to distinguish the different walking impairment levels defined by WISCI II in adult population with iSCI. These results may be explained by several reasons. First, because GDI gait features were originally obtained from 3D kinematic data of children with CP (Schwartz and Rozumalski, 2008), GDI could not be an appropriate index to study gait functionality in adult population with SCI. Therefore, application of the GDI in other pathologies different from CP should be done with caution. Second, WISCI II considers gait impairment in terms of physical assistance, AADs, and orthoses required to walk 10 m, but without providing information concerning joint kinematics related to limb coordination. Thus, other walking ability outcome measures different from WISCI II are necessary to cover the whole functional spectrum of walking ability in population with iSCI, such as categorical and spatiotemporal-related walking and balance measures (Sinovas-Alonso et al., 2021).

This study brings to light the existing lack of scientific literature in relation to an overall gait index that covers the functional diversity of patients with iSCI. This may be due to the fact that gait patterns in iSCI are very heterogeneous and variable depending on the level and severity of the lesion, making it difficult to establish a clear pattern for the set of functional alterations that a subject with iSCI may present. Knowledge of the most commonly altered kinematic variables in iSCI would allow the creation of an overall gait index that could cover the diversity of functional alterations involved in iSCI patients’ gait. This multivariate walking metric would allow a more accurate assessment of the evolution of patients with iSCI by quantifying the changes and, thus, assessing the quality of the therapeutic interventions carried out. Therefore, future work should aim at defining an overall gait index derived from 3D kinematic gait variables appropriate and specific for population with iSCI, focusing on its validation with other walking ability outcome measures.

There are several limitations in our work. The main one is related to the sample size in group with iSCI, which is reduced in some WISCI II levels and not homogeneous between the different levels and neither between iSCI and HV group. This reduced sample is related to the fact that funding lasted for one year of data gathering and we were not able to continue experimentation after July 2021. Sample size was also reduced due to the health situation associated with the COVID-19 pandemic. Furthermore, this research has considered gait maturation at the age of 16 years to ensure that young individuals with iSCI had reach a stable kinematics (Bleyenheuft and Detrembleur, 2012), which restricts the sample size of adults with iSCI included in the study. Another limitation of this study is related to the fact that during 3D kinematic gait analysis individuals with iSCI walked with the minimum external assistance required to walk safely. It means that some of the patients who usually wore orthoses or used canes to walk more comfortably did not use them since the context of the measure was to analyze gait with the least external interferences under medical prescription. It is highly likely that GDI values have been affected by this fact and, consequently, the relationship with the SS WISCI II levels, which were sometimes different from those at the moment of the test. Finally, due to the retrospective design of the study there is a lack of registration of other walking ability outcome measures, what has limited the study to the analysis of the relationship between the GDI and the WISCI II.

The findings of this study indicated that the GDI is not an appropriate multivariate walking metric to represent the deviation of gait pattern in adult population with iSCI from a normal gait profile when it is compared with the levels of walking impairment described by the WISCI II. It is necessary to conduct further research into the development of a new overall gait index derived from SCI-specific 3D kinematic gait variables, involving a larger population, and validating it against other walking ability outcome measures such as categorical and spatiotemporal-related walking and balance measures.

## Data Availability Statement

The raw data supporting the conclusions of this article will be made available by the authors, without undue reservation.

## Ethics Statement

The studies involving human participants were reviewed and approved by Local Ethics Committee of University Hospital Complex of Toledo, Spain. Written informed consent to participate in this study was provided by the participants’ legal guardian/next of kin. Written informed consent was obtained from the individual(s) for the publication of any potentially identifiable images or data included in this article.

## Author Contributions

DH-V, ÁG-A, and IS-A conceived the study. IS-A and AR-G registered data. DH-V analyzed data. IS-A and DH-V wrote the manuscript. RC-d-l-C and AJd-A contributed to the interpretation and discussion of study results. AJd-A obtained funding. All authors revised and approved the final version of the manuscript, read and agreed to the published version of the manuscript.

## Conflict of Interest

The authors declare that the research was conducted in the absence of any commercial or financial relationships that could be construed as a potential conflict of interest.

## Publisher’s Note

All claims expressed in this article are solely those of the authors and do not necessarily represent those of their affiliated organizations, or those of the publisher, the editors and the reviewers. Any product that may be evaluated in this article, or claim that may be made by its manufacturer, is not guaranteed or endorsed by the publisher.
